# Identification of miRs-143 and -145 that Is Associated with Bone Metastasis of Prostate Cancer and Involved in the Regulation of EMT

**DOI:** 10.1371/journal.pone.0020341

**Published:** 2011-05-27

**Authors:** Xinsheng Peng, Wei Guo, Tiejian Liu, Xi Wang, Xiang'an Tu, Dafu Xiong, Song Chen, Yingrong Lai, Hong Du, Guangfu Chen, Guanglin Liu, Yubo Tang, Shuai Huang, Xuenong Zou

**Affiliations:** 1 Department of Orthopaedic Surgery/Orthopaedic Research Institute, The First Affiliated Hospital of Sun Yat-sen University, Guangzhou, Guangdong Province, China; 2 Laura Biotech Co., Ltd. Guangzhou, Guangdong Province, China; 3 State Key Laboratory of Oncology in Southern China, Department of Experimental Research, Sun Yat-sen University Cancer Center, Guangzhou, Guangdong Province, China; 4 Department of Urology, The First Affiliated Hospital of Sun Yat-sen University, Guangzhou, Guangdong Province, China; 5 Department of Surgery, The Second People's Hospital of Zhuhai City, Zhuhai, Guangdong Province, China; 6 Department of Pathology, The First Affiliated Hospital of Sun Yat-sen University, Guangzhou, Guangdong Province, China; 7 Department of Pathology, The First People's Hospital of Guangzhou City, Guangzhou, Guangdong Province, China; Clermont Université, France

## Abstract

The principal problem arising from prostate cancer (PCa) is its propensity to metastasize to bone. MicroRNAs (miRNAs) play a crucial role in many tumor metastases. The importance of miRNAs in bone metastasis of PCa has not been elucidated to date. We investigated whether the expression of certain miRNAs was associated with bone metastasis of PCa. We examined the miRNA expression profiles of 6 primary and 7 bone metastatic PCa samples by miRNA microarray analysis. The expression of 5 miRNAs significantly decreased in bone metastasis compared with primary PCa, including miRs-508-5p, -145, -143, -33a and -100. We further examined other samples of 16 primary PCa and 13 bone metastases using real-time PCR analysis. The expressions of miRs-143 and -145 were verified to down-regulate significantly in metastasis samples. By investigating relationship of the levels of miRs-143 and -145 with clinicopathological features of PCa patients, we found down-regulations of miRs-143 and -145 were negatively correlated to bone metastasis, the Gleason score and level of free PSA in primary PCa. Over-expression miR-143 and -145 by retrovirus transfection reduced the ability of migration and invasion *in vitro*, and tumor development and bone invasion *in vivo* of PC-3 cells, a human PCa cell line originated from a bone metastatic PCa specimen. Their upregulation also increased E-cadherin expression and reduced fibronectin expression of PC-3 cells which revealed a less invasive morphologic phenotype. These findings indicate that miRs-143 and -145 are associated with bone metastasis of PCa and suggest that they may play important roles in the bone metastasis and be involved in the regulation of EMT Both of them may also be clinically used as novel biomarkers in discriminating different stages of human PCa and predicting bone metastasis.

## Introduction

Prostate cancer (PCa) is the most frequently diagnosed malignant tumor and the second leading cause of cancer deaths in western countries [1]. The principal problem arising from PCa is its propensity to metastasize to bone. Skeletal metastases occur in as many as 90% of patients with advanced PCa. Importantly, once tumors metastasize to bone, they are virtually incurable and result in significant morbidity prior to a patient's death [2], [3]. It is very important to understand the mechanism of metastasis formation for preventing metastasis and developing anti-metastatic therapies that may provide additional reduction on the morbidity and mortality of PCa patients.

Skeletal metastasis of tumor is a complicated multi-step process that includes cellular disengagement and motility from the local microenvironment, degradation of the surrounding extracellular matrix, cellular movement, arrested at distal capillaries, extravasate and finally proliferate to form distant secondary bone tumors. All of these processes are regulated by multiple factors and molecular pathways [4]. Although basic knowledge related to this structured process has increased recently, many of the key elements are still poorly understood.

MicroRNAs (miRNAs) are a class of small noncoding regulatory RNAs (19–25 nucleotides) expressed by plants and animals involved in regulation of gene expression. They exert their function by binding to the 3′-untranslated region of a subset of mRNAs resulting in their degradation or repression of translation [5]. Bioinformatic analyses have predicted that single miRNA has multiple targets, and thus miRNAs could mediate the regulation of a great number of protein-coding genes. Recent estimates suggest that one-third of human mRNAs may be regulated by miRNAs [6], [7]. miRNAs have been shown to interfere cellular functions such as cell proliferation, cell differentiation, and apoptosis [8].

Many reports have elucidated the role of certain miRNAs as promoters or suppressors of tumors [9], [10], [11]. An increasing number of observations also gives a collective evidences that miRNAs coordinate some of the intricate gene-expression programs and play a crucial role in tumor metastasis [12]. miRNAs may influence multiple steps of metastatic cascade, such as tumor cell migration, invasion and intravasation. For example, breast cancer is one of the most important contributors of bone metastases [13]. A series of microRNAs have been identified as metastasis promoters, including let-7, miR-9, miR-10b, miR-21, miR-373, miR-520c, and miR-103/107 [12], [14], [15], [16], [17], [18], [19], [20]. Conversely, miR-335, miR-206, miR-31, miR-145, miR-661 and miR-126 have been identified as metastasis suppressor miRNAs in human breast cancer [21], [22], [23], [24], [25], [26], [27].

In PCa, several miRNAs have been identified as mediators of metastasis. It was demonstrated that the deregulation of miR-221 and miR-222 was associated with PCa progression, poor prognosis, and the development of metastasis [28]. miR-21 was also over-expressed in PCa and acts as a key oncogenic regulator that contributes to tumor growth, invasiveness and metastasis [29], [30], [31]. A study has revealed that miR-146a targets ROCK1, and elevated ROCK1 levels promote cell proliferation, invasion and metastasis in the PCa cells [32]. In addition, the genomic loss of miR-101 in human PCa, involved in cancer progression, leads to over-expression of EZH2 [33], [34]. However, the importance of miRNAs in bone metastasis of PCa has not been elucidated to date.

Epithelial-mesenchymal transition (EMT) is a certain signal pathway of describing one key step of the progression of tumor cell metastasis which includes consecutive processes of cell-detaching, migrating, invading, dispersing and final residing [35]. It has been identified as a hallmark of metastasis in multiple tumors, connecting to plenty of transcriptional factors [36], [37], [38], [39]. miRNAs are also components of the cellular signaling circuitry that regulates the EMT program [40]. Recent work has demonstrated several miRNAs, including miR-200 family and miR-205, played critical roles in EMT [41], [42]. Until now, the precise role of miRNAs in regulating EMT is still unclear.

To investigate the role of miRNAs in bone metastasis of PCa and their relationship with EMT, it is firstly need to know miRNA expression profiling in primary and bone metastatic PCa. In the present study, we compared miRNA expression profiles in primary and bone metastatic PCa of humans and identified miRs-143 and -145 related to bone metastasis. Furthermore, we demonstrated that the upregulations of miRs-143 and -145 repressed migration and invasion *in vitro*, tumor development and bone invasion *in vivo*, and EMT of PC-3 cells, a human PCa cell line originated from a bone metastatic PCa specimen.

## Materials and Methods

### Tissue samples

Tissue samples from two groups of PCa patients were studied. Primary PCa tissues were from prostatectomy or transurethral resection in the treatment of local prostate carcinoma. Skeletal metastatic tissues of PCa were from the operation in the treatment of bone metastasis. All samples were formalin-fixed and paraffin-embedded (FFPE) with standard procedures. Regions of tissue specimens >70% cancerous tissue were used for the extraction of total RNA. The histological diagnosis was made by a pathologist and has been re-confirmed by a second pathologist (D.H.). Bone metastasis was diagnosed according to clinical symptom and sign, bone scan, radiography, computed tomography, and MRI. None of the patients had received neoadjuvant hormone, radiation, or chemotherapy before getting the tumor tissues. The clinical information was reviewed about age, bone metastasis, total PSA level, free PSA level and the Gleason score in primary PCa patients. The study was approved by the Institutional Ethical Board (IRB) in the First Affiliated Hospital of Sun Yat-sen University and consented by patients involved.

### RNA extraction

All samples were sent to CapitalBio Corp. and total RNA from FFPE tissue samples was isolated as previously described [43]. In brief, tissue samples were cut into slices from paraffin blocks and placed in 1.5 mL nuclease-free microcentrifuge tubes (Eppendorf), then deparaffinized three times in 1 mL Limonene, followed by wash with 1 mL 100% ethanol twice and air drying at room temperature. Samples were then incubated with digestion buffer (20 mM Tris-HCl, 10 mM EDTA, 1% SDS) and proteinase K (Merck) at 55°C overnight in order to obtain complete digestion of the samples. Subsequently, TRIzol reagent (Invitrogen) was added, and the remainder of the protocol was carried out according to the manufacturer's instructions. RNA samples were resuspended in RNase-free water after the final precipitation step. RNA quality and quantity were assessed using a biophotometer (Eppendorf).

### Microarray analysis

Total RNA samples were analyzed by CapitalBio (CapitalBio Corp.) for miRNA microarray experiments. Each miRNA microarray chip contained 924 probes in triplicate, corresponding to 677 human (including 122 predicted miRNAs), 461 mouse, and 292 rat miRNAs found in the miRNA Registry (http://microrna.sanger.ac.uk; miRBase Release 10.0, 2007). Procedures were performed as described in detail on the website of CapitalBio (http://www.capitalbio.com). Briefly, the low-molecular-weight RNA (LMW-RNA) was isolated using PEG solution precipitation method according to a previous protocol [44]. LMW-RNA was dephosphorylated by Alkaline Phosphatase (NEB) at the first following the protocol given by Wang H, et al. [45]. Then the dephosphorylated LMW-RNA was labeled with 500 ng 5′-phosphate-cytidyl-uridyl-cy3-3′ (Dharmacon) with 2 units T4 RNA ligase (NEB) [44]. Labeled RNA was precipitated with 0.3 M sodium acetate, 2.5 volumes ethanol and resuspended in 20 µl of hybridization buffer containing 3×SSC, 0.2% SDS and 15% formamide. The array was hybridized at 42°C overnight and washed with two consecutive washing solutions (0.2% SDS, 2×SSC at 42°C for 4 min, and 0.2% SSC for 4 min at room temperature). Arrays were scanned with a double-channel laser scanner (LuxScan 10K/A, CapitalBio). The scanning setting was adjusted to obtain a visualized equal intensity of U6 spots across arrays. Data was extracted from the TIFF images using LuxScanTM 3.0 software (CapitalBio Corp). Raw data were normalized and analyzed using the Significance Analysis of Microarrays (SAM, version 2.1, Stanford University, CA, USA) software. Clustering analysis was performed by Cluster 3.0 [46]. All data is MIAME compliant and that the raw data has been deposited in a MIAME compliant database (GEO, accession ID: GSE26964).

### Quantitative reverse transcription-PCR

The cDNA obtained using TaqMan miRNA Q-PCR Detection Kit (GeneCopoeia). Briefly, miRNA was reverse transcribed using sequence specific stem-loop primers (invitrogen) to the following miRNAs: hsa-miR-125b, hsa-miR-145, hsa-miR-153, hsa-miR-210, hsa-miR-143, hsa-miR-100, hsa-miR-363, hsa-miR-451, hsa-miR-572 and hsa-miR-508-5p, based on microarray analysis and their predicted target genes. The reaction was performed with the following parameter values: 15 min at 37°C, 10 minutes at 65°C, 5 min at 85°C, and −20°C until use. Real-time PCR analysis was performed on an iQ5 Real Time PCR Detection System (Bio-Rad) with 20 µL volume reaction containing 2 µL reverse transcription product, 10 µL 2×All-in-One™ Q-PCR Mix, 2 µL PCR Forward Primer (2 µM), 2 µL Universal Adaptor PCR Primer (2 µM), 4 µL ddH2O. The reactions were incubated in 96-well plates at 95°C for 10 min, following by 40 cycles, and then ramped from 66°C to 95°C to obtain the melting curve. Each sample was analyzed in triplicate. No template and no reverse transcription were included as negative controls. U6 snRNA was used as normalization control. Relative expression values from three independent experiments were calculated following the 2^−ΔΔCt^ method of Schmittgen and Livak [47].

### Locked nucleic acid (LNA) in situ hybridization

The procedure was carried out as previously described [48]. Briefly, paraffin-embedded tissue sections were deparaffinized, dehydrated, then treated with proteinase K (20 µg/mL; Roche) at 37°C for 30 min. After washed by 0.2% glycine/PBS for 1 min and fixed with 4% paraformaldehyde, the sections were incubated in hybridization buffer (50% formamide, 5×SSC, 0.1% Tween, 9.2 mM citric acid for adjustment to pH 6.0, 50 µg/mL heparin, 500 µg/mL yeast RNA) at 37°C for 2 h. Digoxigenin-labeled, LNA-modified probes of miR-143 (20 nmol/L; 5′-GAGCTACAGTGCTTCATCTCA-3′, Exiqon) and miR-145 (20 nmol/L; 5′-AGGGATTCCTGGGAAAACTGGAC-3′, Exiqon) were added respectively and incubated at 55°C for 18 h. Sections were washed with 2×SSC twice, then with 2×SSC and 50% formamide at 50°C thrice (30 min each). The anti–DIG-AP (1∶1000, Roche) was added after PBS-T (0.1% Tween 20) wash and incubated at 4°C overnight. Sections were washed four times with PBS-T, and nitroblue tetrazolium chloride/5-bromo-4-chloro-3-indonyl phosphate was used for stain.

### Cell Culture

Metastatic PCa cell lines included PC-3 and LNCaP in the present study. PC-3 was purchased from American Type Culture Collection (ATCC) and maintained in F-12 culture medium (Hyclone) supplemented with 10% fetal bovine serum (Hyclone). LNCaP was purchased from Shanghai Cell Bank, Chinese Academy of Sciences, and maintained in RPMI-1640 culture medium (Gibico, Invitrogen) supplemented with 10% fetal bovine serum (Hyclone). Stably-transfected cells were maintained in media with the presence of puromycin (Sigma-Aldrich). Cells were grown at a humidified atmosphere of 5% CO_2_ at 37°C.

### Generation of Stably Transfected Cell Lines

The sequence of pri-miR-143 and pri-miR-145 were cloned into pMSCV-puromycin plasmid with restriction enzyme Bgl II and EcoR I (New England Biolabs). 293FT cells were then transfected with aforementioned constructed plasmids combined with PIK vector or blank pMSCV-vector as control, using the calcium phosphate method as described previously [49]. After incubation at 37°C for 6 h after transfection, the media were changed and the cells were incubated overnight. To produce new virus, the media were collected thrice a day until 293FT cells reach to total confluence. Viruses are used to infect PC-3 and LNCaP cells. 24 h after addition of viruses, infected cells were selected by adding puromycin to growth medium. Stable cell lines were verified by qRT-PCR. Both pMSCV and PIK plasmids were granted by generous Prof. Song LB, Sun Yat-Sen University Cancer Center, Guangzhou, China.

### Wound healing assay

One day before scratch, stable cell lines of PC-3 and LNCaP cells were trypsinized and seeded equally into 6-well tissue culture plates, and grew to reach almost total confluence in 24 h. When non-serum starvation kept for 24 h after cell monolayer formed, an artificial homogenous wound was created onto the monolayer with a sterile 100 µL tip. After scratching, the cells were washed with serum-free medium. Images of cells migrating into the wound were captured at time points of 0 h, 6 h, 12 h and 24 h by inverted microscope (40×).

### *In vitro* invasion assay

The invasion assay was done by using Transwell chamber consisting of 8 µm membrane filter inserts (Corning) coated with Matrigel (BD Biosciences) as previously described [50]. Briefly, cells were trypsinized and suspended in serum-free medium. Then 1.5×10^5^ cells were added to the upper chamber, whereas lower chamber was filled with medium with 10% FBS. After incubated for 48 h, cells were invaded through the coated membrane to the lower surface, in which cells were fixed with 4% paraformaldehyde and stained with hematoxylin. The cell count was done under the microscope (100×).

### Adhesion assay

The adhesion assay was performed as described previously [51]. Briefly, 96-well plates were coated with 50 µl fibronectin (50 µg/ml) in original media at cell incubator for 1 h. After washed with warm media, the plates were blocked with 1% BSA at 37°C for 1 h and washed twice. After trypsinization, suspended cells were seeded to each well with serum-free media at a density of 1.5×10^4^ cells per well. When incubated the plates for 30 min, non-adherent cells were removed and plates were gently washed twice with PBS. Adherent cells were fixed in 4% paraformaldehyde for 20 min at room temperature, then stained with hematoxylin and counted under inverted microscope (100×).

### Western blotting

For the expression analysis of EMT-related proteins, immunoblotting assay was carried out. All the stable cell lines, including PC-3/vector, PC-3/miR-143, PC-3/miR-145, LNCaP/vector, LNCaP/miR-143 and LNCaP/miR-145, were seeded in 100 mm tissue culture dishes. After 24 h, cells were washed with prechilled PBS when the confluence reached to 60–70%, followed by being harvested in sample buffer [62.5 mmol/L Tris-HCl (pH 6.8), 2% SDS, 10% glycerol, and 5% 2-β-mercaptoethanol]. Equal amounts of protein from the supernatant were loaded per lane and resolved by SDS-polyacrylamide electrophoresis. In sequence, protein was transferred onto PVDF membrane (Millipore), blocked by 5% nonfat milk for 1 h at room temperature, and probed with primary antibodies (1∶1000) for 3 h, including mouse anti-E-Cadherin (BD Biosciences), mouse anti-Fibronectin (BD Biosciences) and mouse anti-Vimentin (BD Biosciences). Membranes were washed thrice (10 min each) in TBS-T buffer and incubated for 40 min at room temperature with horseradish peroxidase-conjugated anti-mouse secondary antibodies. Blots were washed thrice (10 min each) in TBS-T and developed using the ECL system. Protein loading was normalized by reprobing the blots with mouse anti-α-Tubulin antibody (Abcam).

### *In vivo* models of prostate cancer bone metastasis

Intra-tibial injection model was used. ten male severe combined immunodeficient (SCID) mice of 3∼4 weeks old were purchased from HFK Bio-Technology.CO., LTD (Beijing, China). Before inoculation, PC-3 cells were resuspended in 40 µL serun-free F-12 medium at the density of 2×10^5^ cells per 40 µL, and injected with a 26-gauge needle into the tibia using a drilling motion. Animals were randomized into two groups equally, where each 5 animals were treated with PC-3/miR-143 or PC-3/miR-145 on right tibias respectively. All the 10 mice were injected with PC-3/vector on left tibias as self-control. Mice were monitored weekly for tumor growth. On week 5, hindlimbs was radiographed using a Faxitron x-ray machine (Faxitron X-ray Corp, USA) to detect the bone lesions. Then mice were sacrificed, and tibias were collected, decalcified and fixed in formalin for further histologic analysis. Bone lesions were evaluated and calculated as described as previously described [52], where 0 grade for no lesion, 1 for minor lesions, 2 for small lesions, 3 for significant lesions with minor break of margins, and 4 for significant lesions with major break in peripheral lesions.

### Statistical analysis

To determine different expression of miRNAs in Microarray, Significance Analysis of Microarrays (SAM, version 2.1) was performed using two class unpaired comparison in the SAM procedure. Significantly differentially expressed miRNAs was selected as following standards: |Score(d)|≥2 [Numerator(r)/Denominator(s+s0)], Fold Change≥2 or ≤0.5, and q-value(%)≤5 (false discovery rate, FDR) in bone metastasis of PCa compared to primary PCa.

Data were expressed as mean ± standard deviation (SD). Statistics were assessed using SPSS 17.0 (SPSS, Inc., Chicago, IL, USA). In real-time PCR and animal experiments, data were compared by Student *t-test*. The relationship between down-regulated miRNA expression and clinicopathological features in primary and bone metastatic PCa was analyzed using the Spearman rank correlation test. In metastasis assay-based experiments, the data were analyzed with one-way ANOVA. For understanding the relationship between miRNAs, the significant correlations were determined using the kendall rank correlation test. *p*-values of <0.05 were considered significant.

## Results

### miRNA expression profiling between primary PCa and bone metastasis by microarray analysis

To investigate whether miRNAs are differentially expressed in primary PCa and bone metastatic tissues, we collected six matched-pairs of primary and metastatic tissues (from same patient) and compared their expression profiles using a miRNA microarray. Because the total RNA in five pairs of samples was not enough for a microarray experiment, only a matched-pair of samples was successfully performed with a microarray experiment. We observed an obviously increased expression of 18 miRNAs in bone metastasis compared with primary PCa, including miRs-451, -210, -141, -19b, -29b, -16, -20a, -30b, -193a-3p, -15a, -181a, -26b, -200a, -106b, -20b, -486-5p, -15b, -363. The expression of three miRNAs (miRs-145, -143, -612) was obviously decreased in bone metastasis, especially the expression of miR-145 and miR-143 with the reduction of 5.4-fold and 2.7-fold, respectively.

In order to further determine whether the expression of miRNAs had statistically difference in primary PCa and bone metastatic tissues, we compared the miRNAs expression in 6 primary PCa samples and 7 bone metastatic samples using a miRNA microarray. We found that the expression of 5 miRNAs had statistically significant decreased in bone metastasis compared with primary PCa, including miRs-508-5p, -145, -143, -33a and -100 with the reduction of 4.1-fold, 8.1-fold, 5.7-fold, 3.2-fold and 5.3-fold, respectively. No miRNA expression was significantly increased (Table 1).

**Table 1:** Differentially expressed miRNAs identified in bone metastasis of prostate cancer compared to primary prostate cancer by miRNA microarray.

	Gene Name	Score(d)	Fold Change	q-value(%)
downregulated	hsa-miR-508-5p*	2.504157705	4.115822147	0
	hsa-miR-145*	2.467415063	8.056162186	0
	hsa-miR-143*	2.218798715	5.716061535	0
	hsa-miR-33a*	2.061754542	3.179004726	0
	hsa-miR-100*	1.993760316	5.328405946	0
	hsa-miR-153	1.84630048	2.584699735	0
	hsa-miR-125b	1.691829334	4.268859058	0
	hsa-miR-30e	1.664148611	2.168640765	0
	hsa-let-7d	1.663171563	2.445729453	0
	hsa-miR-27b	1.643407131	2.604881765	0
	hsa-miR-615-3p	1.642039074	2.997784318	0
	hsa-miR-363	1.616996368	22.28263337	0
	hsa-miR-801	1.612394022	2.792119805	0
	hsa-let-7a	1.599417634	2.445422202	0
	hsa-let-7e	1.589532583	2.298183277	0
	hsa-miR-19a	1.586018811	2.076116476	0
	hsa-miR-99a	1.585498528	3.717494302	0
	hsa-miR-125a-5p	1.530996885	2.281902276	0
	hsa-miR-23a	1.51606809	2.053798303	0
	hsa-miR-30c	1.510583537	2.340800949	0
	hsa-let-7b	1.464466769	2.664654338	0
	hsa-miR-20b	1.427480317	3.025278364	0
	hsa-let-7c	1.345507222	2.486769965	0.931887
	hsa-miR-325	1.260864694	2.249952437	1.538354
	hsa-miR-106a	1.252346327	2.662764944	1.538354
	hsa-miR-20a	1.189421593	2.939567104	1.78155
	hsa-miR-886-3p	1.089026812	2.72138752	3.071291
	hsa-miR-92a	0.944476573	2.278112416	4.038179
upregulated	hsa-miR-371-5p	−1.911468863	0.160331751	1.538354
	hsa-miR-572	−1.82166417	0.281081268	2.106876
	hsa-miR-210	−1.735311196	0.121967351	4.136671
	hsa-miR-516a-5p	−1.70737646	0.156344976	4.136671

*Significantly differentially expressed miRNAs selected as following standards: |Score(d)|≥2, Fold Change≥2 or ≤0.5, q-value(%)≤5.

**Figure 1:**
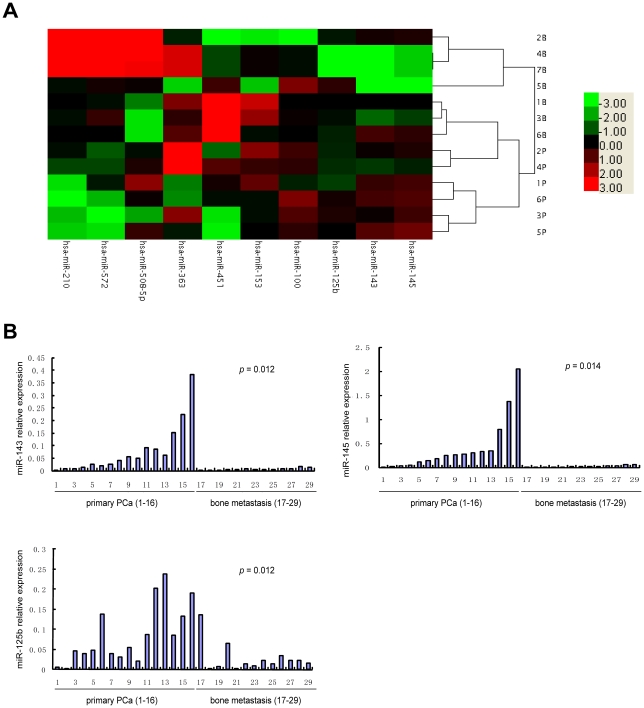
Validation of select miRNAs predicted to be downregulated in prostate cancer. *A,* The certified result of microarray analysis. *B,* Real-time RT–PCR assays on miR-143 (left panel), miR-145 (right panel), and miR-125b (bottom panel) in 16 primary prostate cancer and 13 bone metastasis tissues. The order of the tissue samples is the same for all three plots. *p*-values by *t-test*.

Taken all data together, we have reanalyzed the expression data of 10 miRNAs, including miRs-508-5p, -143, -145, -100, -125b, -153, -210, -363, -451 and -572, by cluster analysis (Figure 1*A*).

### Verification of miRNA microarray data by real-time PCR analysis in primary PCa and bone metastasis

To confirm our microarray data, real-time PCR was performed to analyze the expression of the most significantly regulated miRNAs, including miRs-508-5p, -143, -145, -33a and -100. We examined the expression of miRNAs above from independent samples of 16 primary PCa and 13 bone metastases, which had not been used for microarray analysis. After individual miRNA level in each sample was quantified and normalized to U6 expression, real-time PCR data confirmed that the expression of miRs-145, -143, -33a and -100 with the reduction of 17.3-fold, 12.9-fold, 1.7-fold and 1.7-fold in the bone metastatic tissues, respectively. The expression levels of miRs-143 and -145 were down-regulated significantly in metastasis samples versus primary PCa (*p* = 0.012 and p = 0.014, respectively) (Figure 1*B*). However, the expression of miRs-33a and -100 had no statistic significance (*p* = 0.236 and *p* = 0.448, respectively). miR-508-5p did not express in all primary PCa samples and bone metastatic samples. Although the expression of miRs-125b, -153, -210, -363, -451 and -572 was over 2-fold changes in bone metastasis compared with in primary PCa samples in microarray analysis, there were no statistically significant difference except for the expression of miR-125b with the reduction of 3-fold in the bone metastatic tissues by real-time PCR analysis. This was statistically significant down-regulation in bone metastatic samples (*p* = 0.012) (Figure 1*B*). Thus, the results indicated that there was a significant down-regulation of miRs-145, -143, and -125b when PCa tumors metastasized to bone.

**Figure 2:**
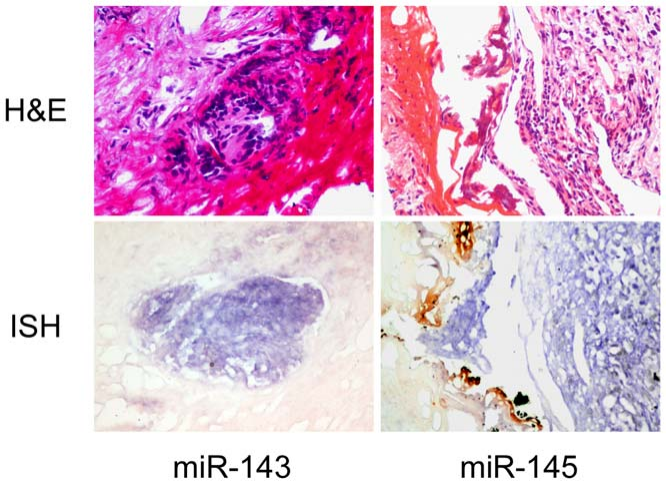
Detection of miRs-143 and -145 in primary PCa tissues by ISH. Sections at upper panel showed the identification of tumor cells and stromal cells by H&E-stainning. Sections at lower panel showed the location of miR-143 (left) and miR-145 (right) in PCa cells by LNA-ISH. Signals of miRs-143 and -145 were in purple blue. Pictures were taken under microscope 200×.

To further identify the major expression sources in primary PCa samples, the LNA-ISH technique was applied. The results showed that miRs-143 and -145 mainly expressed in cancer cells, and their expression in the stromal cells were lower or absent (Figure 2).

### Relative expression of miRs-143 and -145 in the same sample

**Figure 3:**
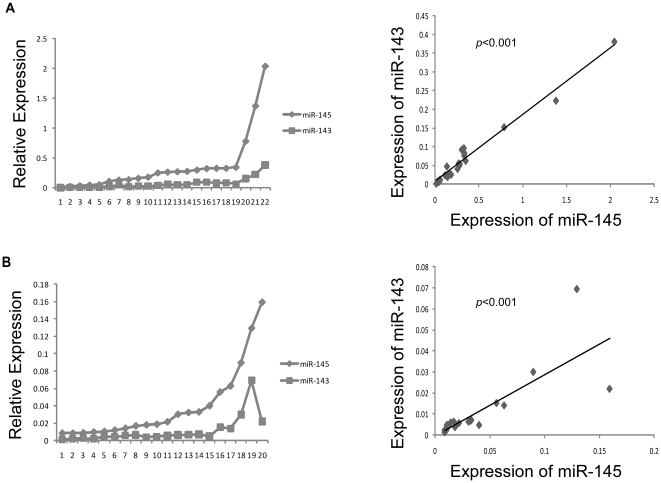
miRs-143 and -145 showed similar expression tendencies in clinical specimens. *A–B,* Expressions of miRs-143 and -145 and their relationships in primary prostate cancer samples (A) and bone metastasis samples (B).

To further investigate whether the expression tendency of miR-145 and miR-143 was identical in the same sample, the relative expression of miR-145 and miR-143 in the same sample was plotted from the real-time PCR in all 22 samples of primary PCa (including 6 microarray samples) (Figure 3*A*) and 20 samples of bone metastases (including 7 microarray samples) (Figure 3*B*), respectively. The significant correlations of miR-145 and miR-143 were found in primary PCa (kendall correlation = 0.850, *p*<0.001) and bone metastases (kendall correlation = 0.765, *p*<0.001).

### Downregulation of miRs-143 and -145 is negatively correlated to bone metastasis, serum PSA level and the Gleason score in primary PCa

**Figure 4:**
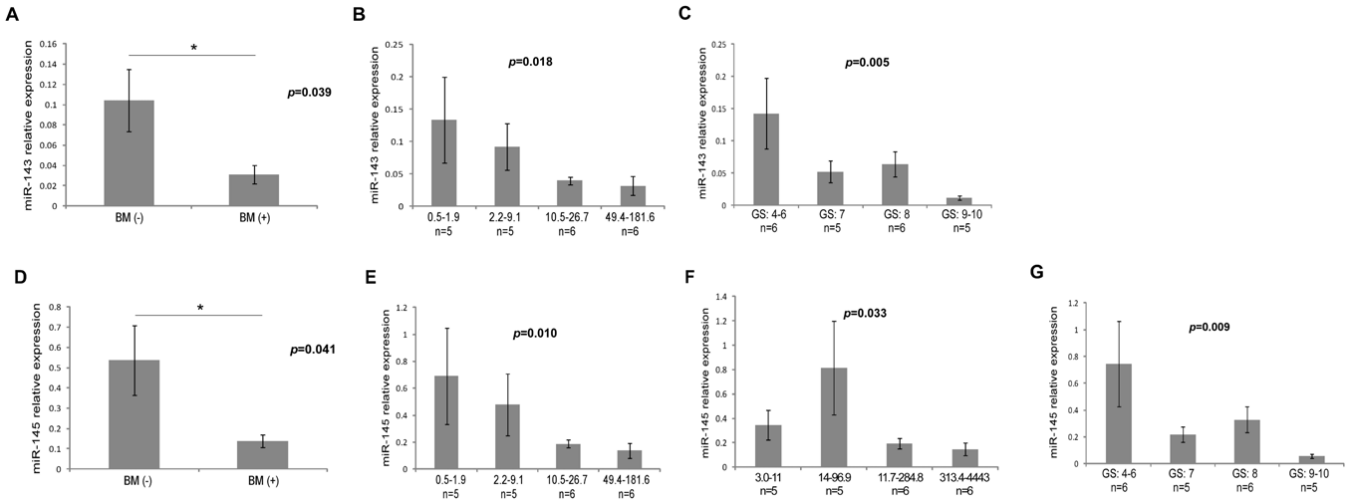
Down-regulation of miRs-143 and -145 is associated with clinicopathological features of primary PCa. *A and D,* The expression of miRs-143 or -145 in the patients with bone metastases was significantly lower than that without metastases (*t-test*, *p* = 0.039, *p* = 0.041, respectively). *B and E,* Tumor samples were divided into four groups with approximately equal sample sizes based on level of free PSA. The level of free PSA in patients with the primary tumor is presented on the x axis. The y axis is the mean of miRs-143 or -145 within each group. The bars represent the standard errors. There was a statistically significant Spearman correlation that characterized an inverse relationship between miRs-143 or -145 expression and free PSA (Spearman correlation = −0.501, *p* = 0.018; Spearman correlation = −0.536, *p* = 0.010). *F,* The level of total PSA is also correlated with miR-145 (Spearman correlation = −0.456, *p* = 0.033). *C and G,* The Gleason scores of primary tumor group are presented on the x axis. The y axis is the mean of miRs-143 or -145 within each group. The bars represent the standard errors. There was a statistically significant Spearman correlation that characterized an inverse relationship between miRs-143 or -145 expression and the Gleason scores (Spearman correlation = −0.574; *p* = 0.005; Spearman correlation = −0.546, *p* = 0.009).

Since we found that miRs-143 and -145 was downregulated in bone metastasis, we postulated that downregulation of miRs-143 and -145 might also be associated with clinicopathological features of PCa patients. Firstly, we performed a retrospective investigation of 22 patients with primary PCa. The results showed 12 patients without bone metastasis and 10 patients with bone metastasis. The distribution of age in 22 patients with and without bone metastases was no significant difference. The expression of miRs-143 and -145 in 10 patients with bone metastases was significantly lower than that in 12 patients without bone metastases (*p* = 0.039 and *p* = 0.041, Figure 4, *A* and *D*). Secondly, we assessed whether the expression of miRs-143 and -145 was related to total serum prostate-specific antigen (PSA) level and free PSA level in primary PCa. The results showed significant inverse correlations between the expression of miRs-143 and -145 and free PSA level (Spearman correlation = −0.501, *p* = 0.018; Spearman correlation = −0.536, *p* = 0.010. Figure 4, *B* and *E*), and a significant inverse correlation between the expression of miR-145 and total PSA level (Spearman correlation = −0.456, *p* = 0.033, Figure 4*F*); whereas no correlation between the expression of miR-143 and total PSA level (Spearman correlation = −0.403, *p* = 0.063). Finally, we investigated whether the expression of miRs-143 and -145 was related to Gleason score in primary PCa. There is also a statistically significant inverse correlation between the expression of miRs-143 and -145 and Gleason score (Spearman correlation = −0.574, *p* = 0.005; Spearman correlation = −0.546, *p* = 0.009, Figure 4, *C* and *G*). These results indicated that downregulations of miRs-143 and -145 were associated with tumor progression and bone metastasis. Downregulation of mir-125b was not correlated to bone metastasis, PSA level and the Gleason score in primary PCa (data not shown).

### Upregulation of miRs-143 and -145 reduced the skeletal aggressiveness of PC-3 cells *in vitro* and *in vivo*

**Figure 5:**
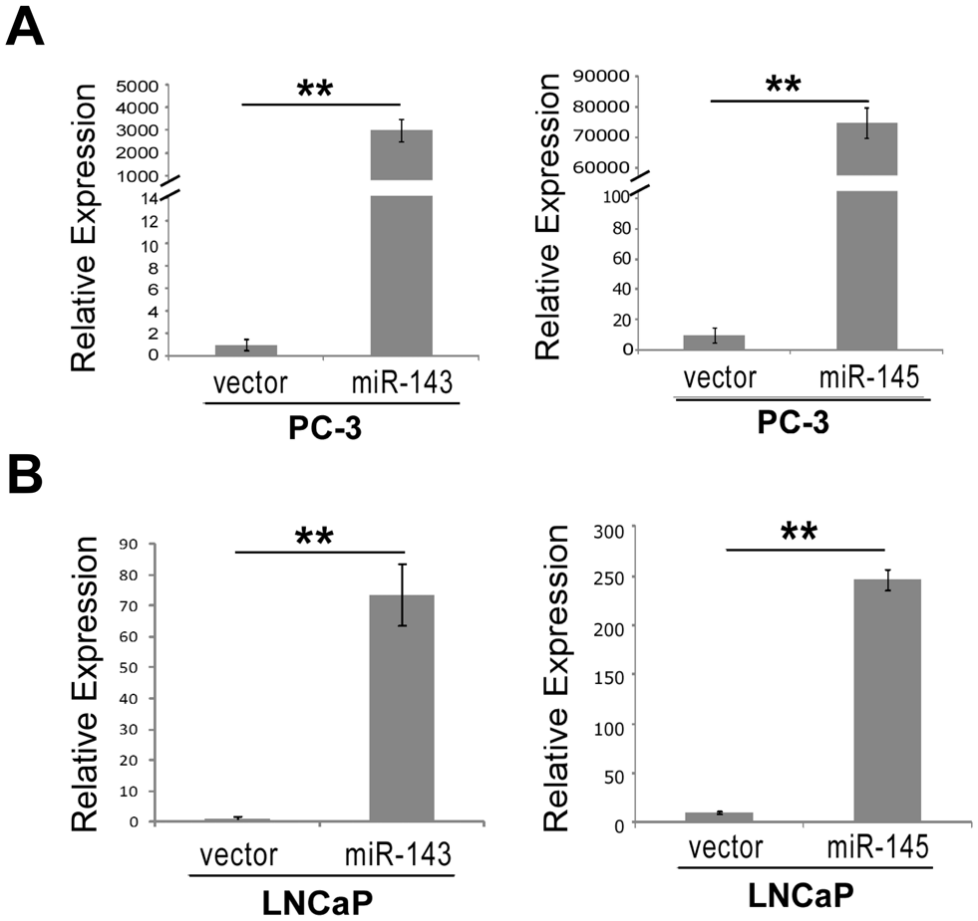
Construction of stable cell lines. *A–B,* Ectopic expression of miRs-143 or -145 in PC-3 (A) and LNCaP (B) were verified by qRT-PCR (*t-test*, *p*<0.01).

**Figure 6:**
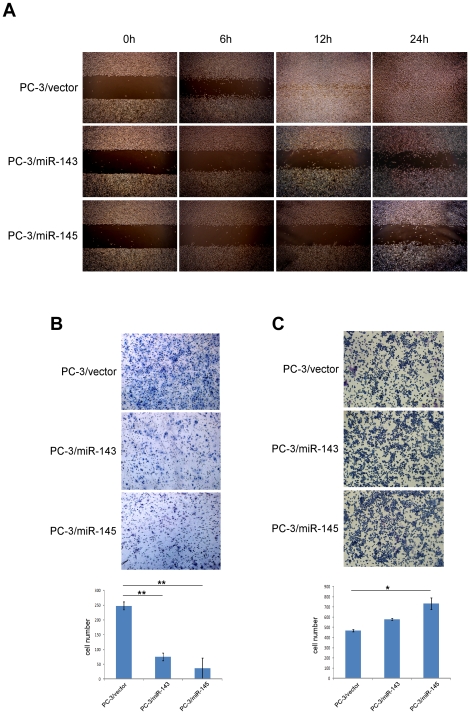
Ectopic expression of miRs-143 or -145 repressed metastasis and increased adhesion of PCa cells. *A,* The migration actions of over-expressing miR-143 were analyzed by wound healing assay. PC-3 cell lines transfected with special plasmids showed significantly different healing conditions. *B,* The invasive properties of indicated cells were tested in invasion assay in a Transwell insert coated with Matrigel. Penetrated cells were counted and analyzed in histogram. *C,* The ability of adhesion of indicated cells were tested in fibronectin-coated plate assay. Adhered cells were counted and analyzed in histogram.

To investigate the role of miRs-143 and -145 in the development and progression of PCa metastasis, miRs-143 and -145 over-expressing cell lines (PC-3/miR-143, PC-3/miR-145, LNCaP/miR-143 and LNCaP/miR-145) were established by retrovirus transfection. Blank plasmid transfected cells, PC-3/vector and LNCaP/vector were used as control groups. As showing in Figure 5, *A* and *B*, fold changes in the relative expression of miRs-143 and -145 transfected PC-3 and LNCaP cell lines were much higher than that these cells transfected with vector (*p*<0.01). Migration, invasion and adhesion assays were performed *in vitro*. Interestingly, cell migration was observed by wound healing assay that it was much slower than PC-3 cells transfected with vector when PC-3 cells transfected with miRs-143 and -145 in a time-dependent manner (Figure 6*A*). The invasive property of PC-3 cells was examined by Transwell-Matrigel penetration assay, which depicted much fewer cells penetrated through the gel-membrane section when PC-3 cells transfected with miRs-143 and -145 than PC-3 cells transfected with vector (Figure 6*B*, *p*<0.01). The invasive property of PC-3 cells was significantly inhibited by miR-143 and -145, even more obviously inhibited by miR-145.

We also examined the effects of miRs-143 and -145 on the adhesion ability of PC-3 cells in order to understand how miRs-143 and -145 affected PCa cells residing to secondary site. The results showed that miR-145 significantly enhanced adhesive ability of PC-3 cells when compared with PC-3 transfected with vector (Figure 6*C*, *p*<0.05). PC-3 cells transfected with miR-143 also presented a higher adhesive ability, but it is not statistically significant. However, LNCaP/miR-143 and LNCaP/miR-145 cells and LNCaP/vector cells did not show significant difference in cell migration, invasion and adhesion (data not shown). Moreover, these results of cell migration, invasion and adhesion indicated that the ability of ectopic miR-145 repressing aggressiveness was more significant than that of miR-143.

**Figure 7:**
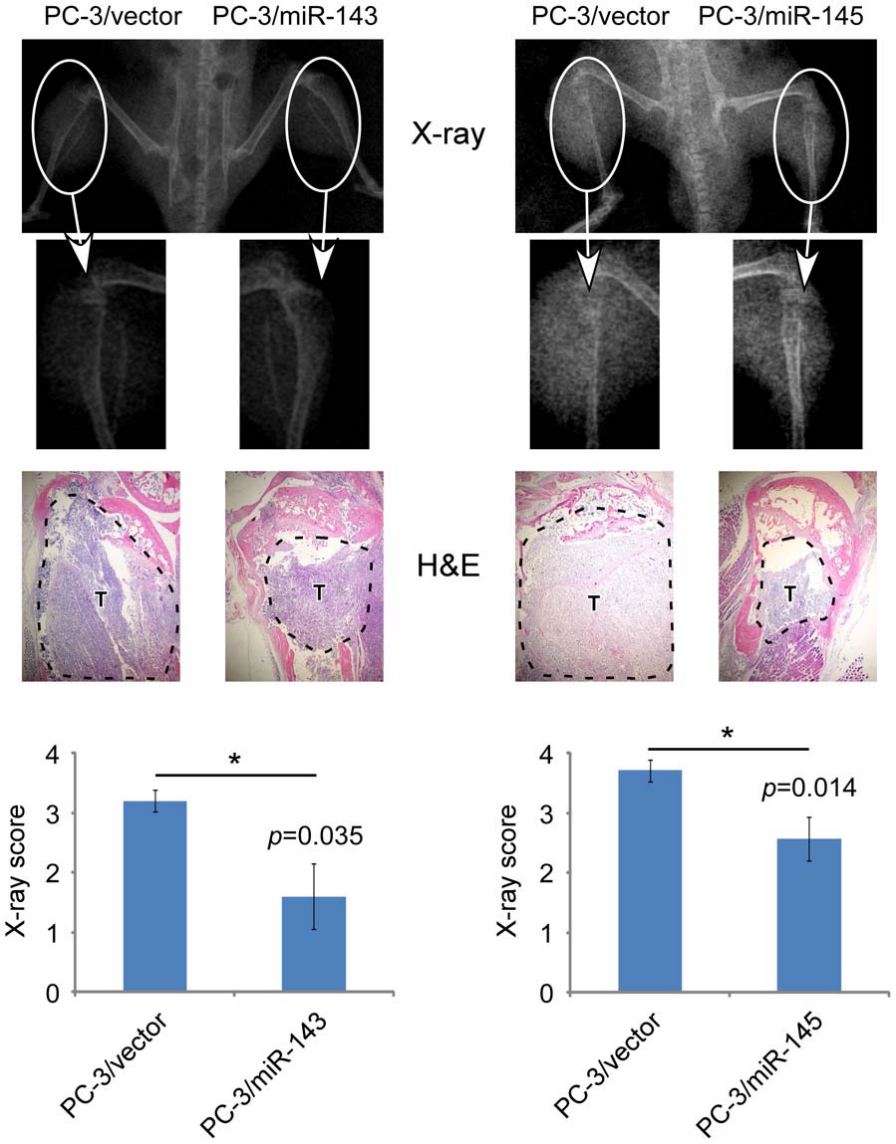
Both of miRs-143 and -145 repressed the development and invasion of PC-3 cells in bone. Male SCID mice were inoculated with PC-3 cells through the intra-tibial route. Skeletal lesions in radiographs are demonstrated by arrows (upper panel), and histologic analysis was carried by H&E-staining in which tumors were lined out by dashed line and marked as “T” (middle panel, taken under microscope 40×). Lesion scores of control and experimental specimens were shown in lower panels, where results were showed by means ± SEM of each group, *p* = 0.035 and *p* = 0.014 respectively.

To further investigate the role of miRs-143 and -145 in the development and progression of PCa metastasis *in vivo*, an intra-tibial injection mouse model was used. Five weeks after intra-tibial inoculation, skeletal lesions of all animals in the left tibias were remarkably larger than those in the right tibias (Figure 7, upper panel), which means PC-3/miR-143 and PC-3/miR-145 had less skeletal invasive ability compared with PC-3/vector. Histological confirmations were made by H&E-stainning (Figure 7, middle panel). The extents and areas of skeletal lesions were assessed by X-ray scores (Figure 7, lower panel), from which PC-3/miR-143 and PC-3/miR-145 revealed to form significantly smaller tumors and bone invasion compared with PC-3/vector (*p* = 0.035 and *p* = 0.014, respectively). The results suggested that miRs-143 and -145 could also repress the development and aggressiveness of PCa in bone.

### Upregulation of miRs-143 and -145 repressed EMT of PC-3 cells

**Figure 8:**
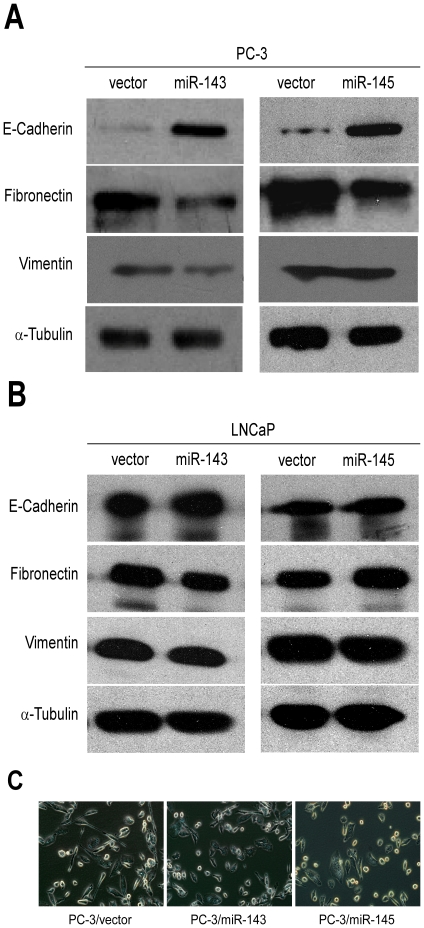
miRs-143 and -145 mediates bone metastasis of prostate cancer via regulating the EMT. *A–B,* The levels of E-Cadherin, Fibronectin and Vimentin were shown in PC-3 (A) and LNCaP (B) cells. α-Tubulin was shown as loading control. *C,* The phenotype of PC-3 cell lines were photographed under microscope (200×).

To investigate whether miRs-143 and -145 regulated bone metastasis by repressing EMT, western blotting analysis was performed for detection of protein expression of E-cadherin, fibronectin and vimentin as described special characteristics of PC-3 and LNCaP cell lines during EMT. The result illustrated that E-cadherin, which is one of epithelial markers and supposed to be down-regulated during EMT, was increased in PC-3 cells transfected with miR-143 or miR-145. Moreover, fibronectin, which is a sort of mesenchymal markers and should be up-regulated during EMT, was repressed in stably expressing miR-143 or miR-145 transfected PC-3 cells, compared to PC-3 cells transfected with vector. However, Vimentin, another mesenchymal marker, was just down-regulated in PC-3 cells when transfected with miR-143 (Figure 8*A*). Nevertheless, all these proteins did not exhibit significant difference in LNCaP cells whatever the cells transfected with miR-143 or -145 (Figure 8*B*).

We tested the ability of miRs-143 and -145 to reverse the mesenchymal phenotype of metastatic PCa cells. PC-3/vector cells are highly invasive and displayed typical fibroblastic morphology, which is in consistent with a very low level of E-cadherin expression. Over-expression of miRs-143 and -145 produced a dramatic shift in morphology, from a stick-like or long spindle-shaped mesenchymal population to a short spindle-shaped or round and flat epithelial population (Figure 8*C*). These suggested that miRs-143 and -145 had negative effects on EMT in PCa and could conduct mesenchymal cells to transdifferentiate toward epithelial cells.

## Discussion

In the present study, the expression levels of miR-143, -145 and -125b were first identified to be down-regulated in bone metastatic tumors compared to primary PCa tumors. Furthermore, primary PCa patients with bone metastasis had significantly lower expression levels of miRs-143 and -145 than those in the patients without bone metastasis. We also found that the expression levels of miRs-143 and -145 were inversely correlated with the Gleason score and PSA level. The upregulations of miRs-143 and -145 reduced aggressiveness of PC-3 cells *in vitro* and *in vivo*, and repressed EMT of PC-3 cells. These results suggest miRs-143 and -145 may play an important role in bone metastasis of PCa.

Downregulations of miR-143 or -145 were found in different tumor types including breast, gastric, liver, lung, bladder, pituitary, ovary and colon [53], [54], [55], [56], [57], [58], [59], [60]. In PCa, miR-143 deregulated in primary cancer compared with normal prostate tissue [61], [62]. The miR-145 also showed lower expression by microarray analysis in primary tumor than in normal prostate tissue [63], [64]. Plenty of studies identified several miR-143 targets including DNMT3A and KRAS [65], [66], as well as miR-145 targets including BNIP3, IRS, C-MYC, YES and STAT1 [10], [67], [68], [69]. These studies also demonstrated both of them as tumor suppressors repressed tumor proliferation or promoted apoptosis.

In a recent study, the processing of miRs-143 and -145 were also involved in metastasis. In microvascuiature, miR-145 expressed in pericytes and repressed the migration of microvascular cells by directly targeting Fli-1 [70]. In breast cancer, miR-145 was identified to suppress cell invasion and metastasis by directly targeting MUC1 [27]. By direct deregulation of FSCN1, miR-145 inhibited invasion of esophageal squamous cell [71]. Furthermore, Sachdeva M, et al. found that miR-145 could target multiple metastasis-related genes including MMP-11 and ADAM-17 [72]. The miR-143 was also demonstrated to abrogate PCa progression in mice by interfering with ERK5 signaling, which is involved in EMT pathway [62], [73]. In our study, the results *in vitro* and *in vivo* both supported that the deregulations of miR-143 and -145 might promote bone metastasis of PCa.

Our study demonstrated that upregulation of miR-143 in PC-3 cells repressed mesenchymal markers of fibronectin and vimentin, and increased E-cadherin, one of epithelial markers. Moreover, up-regulation of miR-145 in PC-3 cells exhibited the same effects on these proteins except for vimentin. Re-expression of miR-143 in SW620 cells of colorectal cancer also increased E-cadherin expression and the cells were in consistent with a transition to a more epithelial-like cell phenotype [60]. These finding indicated that miRs-143 and -145 may be a suppressor of the transition to a more mesenchymal-like phenotype. Given that EMT was considered to be one of the critical steps in tumor invasion and metastasis by allowing cancer cells acquire mesenchymal features that permit escape from the primary tumor [74], E-cadherin plays a critical role as a regulator of signaling complexes and loss of E-cadherin function is a clinical indicator for poor prognosis and metastasis [75]. We can expect that miRs-143 and -145 may inhibit migration and invasion of PC-3 cells by repressing EMT.

Although upregulations of miRs-143 and -145 were able to repress the aggressiveness and EMT of PC-3 cells from bone metastasis, it cannot reverse the metastatic characteristics and regulate EMT markers of LNCaP cells from lymph node metastasis. Especially, deregulation of miRs-143 and -145 was not found in lymph node metastasis comparing to primary PCa tumor with microarray analysis [76]. These findings suggest that miR-143 or -145 may have a cell type–specific function and only inhibit bone metastasis instead of lymph node metastasis, or loss of miRs-143 and -145 could promote the bone metastasis other than lymph node metastasis, which might be regulated by other miRNAs such as miR-221 [76].

Our results also showed a similar expression pattern of downregulated miRs-143 and -145 in a same primary PCa tumor or bone metastasis of PCa. Due to their DNA loci were very close to each other within approximate 2.0 kb at chromosome 5q32 [77] and both precursors might originate from the same primary miRNA [78], we speculate that miRs-143 and -145 could be regulated by some events with a similar mechanism. Moreover, we want to figure out whether one controls the expression of the other one, but there's no study about the interaction between miR-143 and miR-145. Further mechanism should be explored.

miRs-143 and -145 were downregulated to much lower levels in primary PCa patients with bone metastasis, compared with the attenuation in those without bone metastasis. Furthermore, the expression levels of miRs-143 and -145 in primary PCa patients were inversely correlated with Gleason Score, one of the strongest conventional predictors of tumor recurrence [79], indicating higher miRs-143 and -145 expressions might indicate a less possibility of bone metastasis and a better clinical condition, *vice versa*. There is the same relationship between miRs-143 and -145 expressions and free PSA level, one predictor of pathologic stage through clinical stage and biopsy Gleason score [80] and a direct predictor of biochemical progression for PCa-specific mortality [81]. Given the facts above, we expect that the levels of miRs-143 and -145 could be considered as novel biomarkers in discriminating different clinical stages of human PCa and predicting bone metastasis.

A recent study showed that chemically modified miR-143 can be a candidate for an RNA medicine for the treatment of colorectal tumors [82], which could function as anti-cancer drugs in the future. This is a great contribution to a fresh new perspective that can cast light on miRs-143 and -145 as therapeutic targets in bone metastasis of PCa clinically.

In summary, our findings suggest that miRs-143 and -145 may play important roles in the bone metastasis of PCa and be involved in the regulation of EMT. Both of them may also be clinically used as novel biomarkers in discriminating different stages of human PCa and predicting the possibility of metastasis or even as therapeutic targets in bone metastasis of PCa.
